# 753. Impact of a "Real-Time" Case-Based Infectious Disease Elective on Student and Preceptor Perceptions of Confidence with Entrustable Professional Activities

**DOI:** 10.1093/ofid/ofad500.814

**Published:** 2023-11-27

**Authors:** Beth Cady, Kendra Bourland

**Affiliations:** Southern Illinois University at Edwardsville School of Pharmacy, Hillsboro, Illinois; HSHS Saint John's Hospital, Springfield, Illinois

## Abstract

**Background:**

Pharmacy students often struggle transitioning from the didactic to experiential setting, primarily due to the lack of live clinical interaction. A didactic infectious diseases case-based elective was designed to implement this concept (using volunteer preceptors) to create rotation-ready students. The purpose of this project is to evaluate the impact of patient cases on students’ ability to engage in professional, interdisciplinary interactions and to subsequently determine if the students’ perceptions correlate with the evaluation of their performance from volunteer preceptors.

**Methods:**

Third-year pharmacy students were enrolled in the elective and presented with two infectious disease cases throughout the course. Cases required students to interact with an infectious disease pharmacist/resident volunteer acting in the role of an attending physician or microbiology lab technician. Students contacted team members daily (mimicking "rounds") as part of an infectious disease consult service. Students completed each case “in real time” and were tasked with updating the attending physician with appropriate changes to the antibiotic regimen as more information (ie-labs) became available . Volunteer preceptors were surveyed on specific student entrustable professional activities. Students were also asked to self-assess their confidence in these areas. Results from students vs preceptors were then compared.

**Results:**

Of 47 students surveyed (over 2 course iterations), 100% of the students felt confident in their ability to engage professionally with other members of the healthcare team, make evidence-based recommendations, and research complex drug information questions using primary literature at the end of the course. Of 41 preceptors surveyed, > 85% of preceptors felt confident in the students’ abilities to interact professionally, utilize primary literature to make evidence-based recommendations, and answer complex drug information questions.

**Conclusion:**

Student engagement in simulation activities requiring interactions with other healthcare members and research into primary literature leads to confidence in performing essential interprofessional and clinical skills. Preceptor assessment of these skills was in agreement with what students perceived.

**Disclosures:**

**All Authors**: No reported disclosures

